# Effect of counselling intervention on perception of stressful school event among educational administrators and special educators: Implications for medical educational policy

**DOI:** 10.1097/MD.0000000000051421

**Published:** 2026-11-06

**Authors:** Theresa Chinma Agodo, Ngozi Sandra Ikenyesi, Vera Nneka Ogakwu, Mercy Benedette Ifeoma Uba, Ogochukwu Vivian Nwabuani, Florence N. Ogbonna, Patrick Chinenye Okafor, Moses Onyemaechi Ede, Beth Nnenne Oluka, Nneka Nwosu, Andrew Nnabuchi Ogbochie, Ndubuisi Collins Enyi

**Affiliations:** a Department of Educational Foundations, Faculty of Education, University of Nigeria; b Department of Sociology, Delta State University, Abraka, Nigeria; c Department of Guidance and Counselling, Faculty of Education, Alex Ekwueme Federal University, Ikwo, Ebonyi, Nigeria; d Department of Continuing Education and Development Studies, Faculty of Education, University of Nigeria, Nsukka; e Department of Educational Foundations, Odumegwu Chukwuemeka Ojukwu University, Igbariam, Anambra, Nigeria; f Department of Special Education, Ebonyi State University, Abakaliki, Ebonyi, Nigeria; g Department of Educational Psychology, Faculty of Education, University of Johannesburg, South Africa; h Department of Counselling and Human Development Studies, Faculty of Education, University of Nigeria, Nsukka; i Department of Administration and Local Government, University of Nigeria, Nsukka; j St. Augustine Catholic Church, Manso Amenfi, Ghana.

**Keywords:** administrators, CBT, counselling intervention, nonspecialist teachers, special education, work stress. medical education

## Abstract

**Background:**

The physiological and psychological demands and responses in the workplace, including school settings, are very high among school administrators and teachers. They experience high levels of stress due to several factors, such as heavy workloads, emotional and psychological pressures, and challenging working environments. Consequently, this has a continued negative impact on their job performance and productivity. Some individuals may experience this negative impact more than others based on how they perceive stressful situations. With this in mind, this study aimed to investigate the effect of counseling intervention on the perception of stressful school events among professional educational administrators and special educators in Enugu State, Nigeria.

**Methods:**

The study involved 85 administrators and nonspecialist special educators who were divided into 2 groups: 43 participants in the experimental group and 42 in the control group. In Nigeria, a quasi-experimental design was chosen and carried out. The Teachers’ Job Stress Scale was used to quantify the special educators’ occupational stress at 3 different stages. Cronbach’s alpha (0.79) coefficient was used to verify the scale’s validity and reliability. Analysis of covariance was used to examine the gathered data.

**Results:**

The study results indicate that counseling intervention was effective in reducing stress among administrators and nonspecialist teachers in special schools in Enugu State. The findings also suggest that time significantly impacts administrators’ and nonspecialist teachers’ work-related stress. This implies notable differences in participants’ reactions to work-related stress at different points in time. Follow-up results indicated that the intervention’s effectiveness persisted over time. Gender did not have a statistically significant impact on participants’ occupational stress.

**Conclusion:**

The study concluded that REBT is a therapeutic stress management strategy that is significant in the reduction of work stress among Agricultural and Industrial Arts Educators. Therefore, university administrators should establish a REBT institute where the mental health of staff members will be frequently monitored.

## 1. Introduction

Administrators are those responsible for managing, directing, organizing, and evaluating public affairs of institutions. Administrators working in educational institutions are called educational administrators. Administrative roles in managing the affairs of educational institutions are very demanding. The various demands emanating from administrative roles could be regarded as administrative stress. Stress is one of the public health challenges that affects everybody, including educators. It has health and psychological dimensions. The health dimension is when the human body reacts to excess workloads to protect the heart from threats.^[1]^ The hypothalamus is a small area at the base of the brain that triggers the body’s alarm system when you perceive a threat.^[1]^ A big dog barking at you on your morning stroll is an illustration of a felt threat. This system triggers the adrenal glands, which are located above the kidneys, to generate a spike in hormones like cortisol and adrenaline through nerve and hormonal impulses.^[1]^ On the other hand, the psychological dimension shows that stress is behavioral. It is a typical psychological reaction to life’s demands and obstacles.

It poses so many threats to people, including teachers and administrators across the globe. Researchers described the teaching profession as a very stressful job.^[2,3]^ In the Nigerian classroom setting, teachers face excessive occupational stress.^[4]^ In recent years, teachers’ jobs have become increasingly stressful, which tends to lower their efficacy as teachers in Nigeria.^[5]^ According to past studies, Nigerian teachers experience high levels of stress.^[6,7]^ Stress has a greater tendency to dehumanize individuals who work with students who have special needs.^[8–10]^ Teachers of children with special needs are more likely to report feeling overwhelmed than teachers of normally developing or disabled children.^[11,12]^ This could be due to labor requirements associated with the disabilities of children on the spectrum. For example, teachers of students with ASD must complete additional tasks such as modifying the classroom environment and curriculum to meet the students’ needs.^[12,13]^ In the same vein, teachers are experiencing various levels of stress. According to Brown and Nagel,^[14]^ teachers often feel more job-related stress than professionals in other disciplines. Not only Nigerians, but stress also affects most individuals worldwide, yet it varies from nation to nation.^[15]^ For instance, according to Ipsos, 76% of Turkish respondents said they felt worried, compared to 44% in Japan.^[15]^ According to a South African study, there were no significant differences in the stress levels of instructors among the various institutions.^[16]^

Nonspecialist teachers in special schools are more likely to experience stress and burnout because they must adapt to new expectations and need new resources, particularly new professional skills. Due to increased responsibilities associated with a population that is typically more challenging to motivate, teach, and evaluate,^[16,17]^ the introduced changes necessitate new educational practices, which cause stress for teachers working with children and youth with disabilities.^[18,19]^ They were overstressed by the pupils’ needs and the responsibility, and they felt disdained and without power.^[20]^ Also, Stempien and Loeb^[21]^ studied 116 teachers of regular education and special education and found that special education teachers were the most unsatisfied and frustrated group.

How people interpret stress-induced events could be either maladaptive or adaptive. Some workers, especially professionals in education settings, rarely have accurate and realistic perceptions about school events. For example, I am the only person who receives more workload daily. If it continues that way, I must resign, getting tired of administrative workloads. This type of thinking is negative, but if individuals are aware of resilience skills, they have the opportunity to reconsider. In other words, resilience and stressful events are connected.^[22]^ Research indicates that the more teachers feel threatened or lose something, the more emotional exhaustion they experience, leading to poorer well-being and difficulties managing students. This can result in students feeling alienated, confused, failing in school, and dropping out.^[23,24]^

Seibt et al^[25]^ found significant differences based on gender, with women showing higher stress levels, and educational attainment, with less educated instructors experiencing higher stress levels. However, other authors have not confirmed these findings. For example, Esteras et al^[26]^ reported higher stress levels among male teachers. Similar research was done with 67 special education teachers by Küçüksüleymanoğlu,^[27]^ who found variances according to gender and educational level. Men reported higher degrees of emotional exhaustion and depersonalization, whereas women reported lower rates of personal and professional success. Teachers with greater levels of education were more likely to feel emotionally exhausted, even though there were no differences in educational achievement.

Individuals who exhibit irrationality towards stress may also misinterpret challenging situations in school. Globally, school populations are diagnosed with mental health issues.^[28]^ where individuals with depression often hold negative automatic thoughts about themselves, the world, and the future.^[29]^ Healthy negative emotions are linked to rational beliefs and adaptive behaviors, while unhealthy negative emotions are associated with irrational beliefs and maladaptive behaviors.^[30,31]^

Therefore, we believe that implementing Counseling intervention conceptualized in this study as cognitive behavioral therapy (CBT), which aimed to reduce job stress among administrators and nonspecialist teachers in special schools in Enugu state, Nigeria, would be effective. CBT would serve as the theoretical basis for this intervention. CBT is a form of psychotherapy that helps patients recognize and change harmful thought patterns affecting their behavior and emotions.^[32]^ By encouraging more balanced thinking, CBT can enhance stress management skills and help individuals become more optimistic and better problem solvers under stress.

CBT emphasizes that a person’s emotional experience is influenced by their understanding of the events leading up to that experience.^[33,34]^ Through behavioral experiments and cognitive restructuring, cognitive therapies aim to promote more adaptive thinking by identifying and addressing biased thought patterns. Cognitive restructuring helps patients recognize and challenge thinking traps, leading to more flexible and realistic interpretations of events.

Cognitive Behavior Therapy provides the foundation for cognitive behavior-aided counseling, with several studies demonstrating its benefits.^[35,36]^ CBT is an evidence-based therapy that focuses on changing beliefs and behaviors to improve emotional well-being.^[37]^ It is effective in treating various psychological issues, including stress.^[38]^ By helping individuals manage stress and overcome negative thoughts, CBT can improve overall well-being and happiness, both at work and in personal life.

CBT can help teachers effectively manage stress, improve their daily lives, and enhance the quality of education and administrative services they provide. Research on the use of cognitive-behavioral counseling for stress management in special education settings is limited, particularly in Enugu, Nigeria. Further studies are needed to explore the impact of cognitive-behavioral counseling on work stress among administrators and nonspecialist teachers in special schools.

On one hand, nonspecialist teachers in special schools are those teaching staff who are not professionally trained as special educators. On the other hand, administrators are those managing the human, material, and financial resources in special schools. Administrators are essential in special education because they ensure that regulations are followed, supervise programs and services for children with disabilities, and assist staff and students. Special education administrators oversee the special education team in a school system or particular school, which frequently consists of instructional assistants, special education instructors, and administrators. The role of special education administration is to help parents, instructors, and students enrolled in special education programs by keeping an eye on all current special education funding, regulations, and programs. Teachers often require support from special education administration to set learning objectives and conduct meetings with children and their families. After setting the students’ goals, the administration assists the instructor in delivering education.

Additionally, the administration team helps the instructor adhere to all special education regulations. Teachers in most schools receive an annual performance review from the administrator. Parents enrolling their child in a special education classroom receive assistance from the special education administration to understand their legal parental rights and available resources to support their child. During meetings between instructors and parents, the administrator acts as a mediator and provides families with activities to do at home.

These activities place physiological and psychological demands on special education administrators in Nigeria. Many of them have been hospitalized, suffering from various medical and psychological problems, yet little to no support has been given to them. Often, both teachers and administrators carry out official duties at home and are expected to assist students with disabilities, which can lead to work/family conflict and increase the risk of burnout. This frequently prevents people from effectively balancing their home and work responsibilities, resulting in role conflict and physical stress. The core of professional stress and burnout is emotional weariness, which can stem from the tension administrators and instructors accumulate from their jobs, health, and homes. This can lead people to form illogical opinions that negatively impact their well-being and productivity. Despite the prevailing health situation, interventions addressing stress-related problems among special education administrators and nonspecialist teachers in sub-Saharan Africa are very limited. Closing this gap is necessary to assess the effectiveness of interventions tailored to this particular demographic. Members of this group could benefit from more specialized and situation-specific mental health treatments once this gap is addressed. Against this backdrop, the current study examined the effectiveness of counseling intervention on work stress among administrators and nonspecialist teachers in special schools in Enugu State, Nigeria. The study hypothesized that there would be a significant difference in work stress reduction between participants in the counseling intervention and those in the control group at posttest and follow-up stages. Additionally, it was expected that there would be no significant interaction effect between counseling intervention and gender on the mean stress management scores of special education administrators and nonspecialist teachers.

## 2. Research method

### 2.1. Ethical approval

Permission to conduct this study was approved by the lead researcher’s institution, as in the American Psychological Association.

### 2.2. Participants recruitment and study procedure

Participants were recruited from various schools by the research team. Each administrator and special educator received an invitation to participate, which included informed consent, the option to withdraw at any point, and a guarantee of confidentiality. A total of 111 individuals who expressed interest were screened for eligibility based on specific criteria.

Inclusion criteria consisted of being willing to participate, holding an administrative position in a special school or office, being a teacher in a special school, agreeing to participate either in writing or verbally, and scoring above a certain threshold on a scale. Exclusion criteria included active involvement in any pharmaceutical, non-pharmaceutical, pharmacological, or non-pharmacological treatments. The research team also excluded individuals who were not affiliated with a special school or did not meet the inclusion criteria.

Out of the 111 school administrators and teachers who were screened, 85 met the eligibility requirements and were recruited as participants for the study. The eligible participants were presented with 83 cutout papers in 2 colors yellow and red colors, by the research team. The yellow colors were 43 cutout papers, and the red colors were 42 cutout papers. This was to conceal and blind the process of the randomization. Each participant was asked to pick a cutout paper. Those who selected yellow papers were assigned to the treatment group, while the counterpart was assigned to the control group. A sequence allocation method was adopted by the research team to assign group 1 participants and group 2 participants to respective arms (treatment and control groups).

Given the 85 administrators and special educators, the researchers further checked its adequacy to confirm if the power of sample size was good enough for the present study. The Gpower statistical tool showed 0.86 using the following parameters.

The members of the treatment arm received counseling intervention^[39]^ Session 1 focuses on introduction, establishment of therapeutic rapport, guiding norms, and meaning of stress. The second session’s topic was the reasons why teaching can be stressful. The third session focused on the consequences of stress on educators and how it affects inclusive educators. The idea of a cognitive behavioral intervention as an activating event was explored in the fourth session. The discussion of dysfunctional ideas pertaining to teachers’ stress was the main topic of the fifth session. Based on the prior lesson, the sixth session concentrated on group interaction. The therapeutic program came to an end in session 8, while session 7 focused on more hands-on activities, including the usage of logical words in speech. The treatment lasted for 8 weeks, followed by a one-week follow-up assessment after 2 months, which led to the third assessment (Time 3).

### 2.3. Cognitive-behavior-aid counseling program

The counseling intervention used in this study was adapted from Ubah et al^[39]^ which aimed at stress reduction. Before the study was published, the present researchers were privy to the treatment guidelines and used it. The training material is intended to correct the cognitive mistakes and address dysfunctional beliefs accountable for school stress. Additionally, to assist special education teachers in developing fundamental cognitive abilities and stress-reduction strategies. The handbook includes many of the procedures used by the therapist throughout the intervention, such as cognitive restructuring, group cognitive interaction, and cognitive self-report evaluation techniques. The manual covered 8 areas, which included familiarization, understanding and conceptualizing the problem, as well as how the problem originated. Additionally, it included understanding how to deal with the problem (stress), the role of cognitive behavioral counseling strategy in dealing with stress, group interaction, practical exercises on the use of logical words in speaking, and the termination of the therapeutic programme.

The control group members were exposed to a normal school counseling programme (conventional method). The control group participants received a placebo, that is, normal routine school counseling that was not based on cognitive behavior therapy. The counseling, which focused on anger management, lasted 8 weeks, and with our research team’s help, their school counselor was able to complete it in 8 sessions.

### 2.4. Integrity protection

The researchers hired 2 research assistants in order to preserve the integrity that is advised in experimental studies.^[40–46]^ The research assistants had 4 to 6 years of experience in counseling. Both were responsible for monitoring the implementation processes of the intervention. Each person was assigned to the intervention group and the other to the control group. They were equally briefed about the program by the research team.

## 3. Design

This study employed a quasi-experimental design, specifically a nonequivalent control group pretest/posttest design.

### 3.1. Dependent measure

Teachers Job Stressor Scale (TJSS): The TJSS is a brief instrument consisting of 23 items that was developed by Naono-Nagatomo, Abe, and Yada.^[47]^ Its purpose is to measure stressors among inclusive secondary school teachers in Enugu state. The scale was weighed as follows: strongly agree = 4 points, agree = 3 points, disagree = 2 points, and strongly disagree = 1 point. The scale’s appropriate Cronbach’s alpha coefficient, which ranges from 0.7 to 0.87, confirms that the scale’s 5 identified elements exhibit sufficient internal consistency,^[47]^ and the reliability (0.79) of the instrument was confirmed in this study.

### 3.2. Method of data analysis

Data were analyzed using both descriptive and inferential statistics. Mean, standard deviation, and repeated measures of Analysis of Variance statistical method were employed. The sphericity assumption was considered and found to be broken based on Mauchly’s tests [x^2^(2) = 17.722, *P* < .001, ε=855] as determined by TJSS. In order to analyze the 2 TJSS levels for being more than 0.75, we applied the Huynh-Feldt method. To examine the follow-up data, a univariate analysis was used. Individual demographics were analyzed using frequency, percentage, and Chi-square.

## 4. Results

In Table 1, the demographic information demonstrated that Gender (*χ*2 = 0.107, *P* = .744), marital status (*χ*2 = 0.825, *P* = .662), work experience (*χ*2 = 1.065, *P* = .587), academic status (*χ*2 = 1.797, *P* = .180), treatment group, and waitlisted control group did not significantly differ from each other. Participants in the treatment group and waitlisted control group had significantly different educational backgrounds (*χ*2 = 7.953, *P* = .019).

**Table 1:** Descriptive statistics for the socio-demographic characteristics of participants.

Variable	Categories	Intervention group	Controlled group	x2	*P*
Gender	Male	22 (52.4%)	20 (47.6%)	.107	.744
	Female	21 (48.8%)	22 (51.2%)		
	Single	7 (41.2%)	10 (58.8%)		
Marital status	Married	28 (53.8%)	24 (46.2%)	0.825	.662
	Divorced	8 (50.0%)	8 (50.0%)		
Educational qualifications	NCE	16 (61.5%)	10 (38.5%)		
	Bachelor degree	25 (54.3%)	21 (45.7%)	7.953	.019
	Master degree	2 (15.4%)	11 (84.6%)		
Years of experience	Below 10 yr	13 (54.2%)	11 (45.8%)		
	10 to 20 yr	20 (54.1%)	17 (45.9%)	1.065	.587
	Above 20 yr	11 (41.7%)	14 (58.3%)		
Academic status	Administrator	16 (61.5%)	10 (38.5%)		
	Non specialist teachers	27 (45.8%)	32 (54.2%)	1.797	.180
	Above 8 h	17 (39.5%)	19 (44.2%)		

**Keys:** NCE = Nigeria Certificate Education, *P* = significance, *χ*2 = chi-square.

Results in Table 2, *F* (1,81) = 101.076, *P* < .01, *d* = 2.65, *η*^2^p = 0.56, demonstrate a significant decrease in work stress among administrators and nonspecialist instructors who were exposed to the intervention. Additionally, *F* (1.848, 149.699) = 26.189, *P* < .01, *η*^2^p = 0.244 indicates a statistically significant influence of time on work stress among administrators and nonspecialist teachers. This suggests that there are notable differences in the individuals’ reactions to work stress at various assessment dates. Additionally, the results show that the group and time interaction effect did not substantially affect the participants’ job stress scores (*F* [1.848, 149.699] = 69.090, *P* < .01, η^2^p = 0.460). According to the follow-up results, the intervention’s efficacy was sustained over time (*F* [1,81] = 93.055, *P* < .01, *d* = 2.09, *η*^2^*P* = 0.51).

**Table 2:** A repeated measure analysis of variance on the effect of the intervention on stress.

Outcome	Time	Intervention mean (SD)	Control mean (SD)	*F*	Group effect			Time			Time * Groups		
					*P*	*d*	*ηp* ^*2*^	*F*	*P*	*ηp* ^*2*^	*F*	*P*	*ηp* ^*2*^
TJSS	1	75.16 (3.18)	75.59 (2.89)										
	2	68.23 (3.84)	77.20 (2.87)	101.076	.000	2.65	0.56	26.189	.000	.244	69.090	.000	.460
	3	71.29 (2.96)	77.40 (2.87)	93.055	.000		0.51						

Keys: *d* = Cohen’s d, *F* = F-ratio, *P* = significance, TJSS = teacher job stress scale*, ηp*^*2*^ = partial eta square.

The results in Table 3 show there is no statistically significant influence on participants’ gender with regard to work stress, *F* (1, 81) = 1.392, *P* = .242, *η*^*2*^_*p*_ = 0.017. It also indicated that work stress among participants’ scores was not significantly influenced by the group and gender interaction effect, *F*(1,81) = 7.149, *P = *.009, *η*^*2*^_*p*_ = 0.081. Gender did not have a significant effect on the career-related stress experienced by the participants. Furthermore, the interaction between group and gender did not significantly influence how participants assessed their work-related stress levels.

**Table 3:** Moderating and interaction effect of gender and group by gender.

Source		Intervention mean (SD)	Control mean (SD)	*F*	*Sig.*	
Gender				1.392	.242	.017
Groups * Gender	Males	68.28 (3.86)	76.20 (2.68)			
				7.149	.009	.081
	Females	68.18 (3.91)	78.11 (2.79)			

There was a significant difference between Time 1 and Time 2, according to Sidak’s post hoc analysis in Table 4 for the effect of Group according to Time (Mean difference = 2.651, standard error = .420, *P* = .01, CI = 1.628, 3.674). From Time 2 to Time 1, a similar difference was seen (Mean difference = −2.651, standard error = .420, *P* = .01, CI = −3.674, −1.628). Furthermore, the participants’ scores at Time 3 remained considerably lower than those at Time 1 (Mean difference = −1.030, standard error = .381, *P* = .025, CI = −1.959, −.100).

**Table 4:** Posthoc analysis of time as measured by TJSS.

(I) Time	(J) Time	Mean Difference (I-J)	Std. error	Sig.	95% Confidence interval (lower bound, upper bound)
1	2	2.651	.420	.000	1.628, 3.674
	3	1.030	.381	.025	.100, 1.959
2	1	−2.651	.420	.000	−3.674, -1.628
	3	−1.621	.296	.000	−2.343, -.900
3	1	−1.030	.381	.025	−1.959, -.100
	2	1.621	.296	.000	.900, 2.343

## 5. Discussion and conclusion

The results of the study showed that cognitive-behavioral intervention was effective in reducing stress among administrators and nonspecialist teachers in special schools in Enugu state. The findings also suggest that time has a statistically significant impact on administrators’ and nonspecialist teachers’ work-related stress. This implies notable differences in participants’ reactions to work-related stress at various points in time. Additionally, the results show that the group and time interaction effects had no significant impact on participants’ scores related to work stress. Follow-up results indicate that the intervention’s efficacy persisted over time. Gender had no statistically significant impact on participants’ occupational stress. The group and gender interaction effects also had no significant impact on participants’ ratings related to work stress. Post hoc analysis confirmed that the intervention is a useful and successful tactic for lowering stress levels among administrators and nonspecialist teachers.

These results align with earlier research showing that teachers’ occupational stress is decreased by cognitive-behavioral intervention.^[48]^ They also support the findings of Ezegbe et al^[38]^ who found that cognitive-behavioral intervention significantly reduces anxiety and depression in social science education students compared to a waitlisted group. Ejikeme et al^[49]^ found that individuals in the immediate intervention group experienced a substantial reduction in stress symptoms and perceived stress compared to those in the control group. The rational emotive health education program successfully lowered stress levels and negative beliefs regarding education for those teaching personnel who participated in the intervention.^[50]^ Additionally, REBT was effective in decreasing and managing occupational stress.^[49]^

Parents of children with mental retardation may find that the cognitive-behavioral approach is an evidence-based treatment for reducing stress, anxiety, and depression.^[51]^ The results also support the effectiveness of rational-emotive group coaching in reducing stress symptoms in safety officers who receive psychological treatment.^[52]^ This demonstrates the success of cognitive-behavior-assisted counseling and its universal applicability. The study suggests that cognitive-behavior counseling is the most effective method for resolving stress problems among special education teachers.

Regarding gender, the results align with a study by Nwimo and Onwunaka^[6]^ that found male instructors in special schools experience higher levels of stress than females. The findings also support the findings of Wang et al^[53]^ who found that males had higher rates of stress responses to psychological stress than females. However, gender disparities were noted by Addison and Yankyera,^[54]^ who found that women are more stressed than men. The impact of gender on workers’ stress levels may differ due to physical attributes, as suggested by Miller.^[55]^ This could be due to the reason that stress affects both males and females. But to some extent, males may experience more stress than their female counterparts. In this vein, the results from different studies could mean that females could have more stressful experiences compared to their male counterparts. By indication, there should be a consistent stress management strategy in special education settings, which is manned and coordinated by the school counselors. Taking this step would support constant checks on the well-being of workers in workplaces. The findings indicate that counselling intervention using cognitive behavior therapy is one of the best strategies to resolve stress cases among administrators and nonspecialist teachers in special schools due to its evidence-based efficacy.

## 6. Implications

For educational implications, administrators and teachers are critical stakeholders in educational decision-making, and policy-making should strategize how special schools could have well-equipped counseling centers. By doing this, the centers will serve as rehabilitation and counseling centers where teachers with chronic stress and other mental illnesses can be assisted.

The teaching profession can be extremely demanding, and this stress can result in poor work performance, burnout, and decreased job satisfaction. When stress becomes chronic, it is a risk factor for various mental diseases, including anxiety and depression, and can interfere with daily functioning and emotional equilibrium. By including counseling interventions with a specific emphasis on cognitive behavior therapy, medical professionals such as physicians, nurses, and therapists can greatly improve stress reduction programs for educators.

This entails creating counseling centers that are teacher-friendly in special schools, offering evidence-based counseling strategies, and customizing interventions to address the unique challenges teachers face. Medical education professionals can learn more about the unique pressures teachers encounter, such as workload, difficulties managing the classroom, and a lack of resources, by collaborating with school systems to conduct surveys and focus groups. This knowledge will help them understand counseling strategies such as cognitive behavior therapy, and assist educators, including administrators, in recognizing and disputing harmful ideas and attitudes that fuel stress. Working together with current school personnel to guarantee a unified strategy for mental health assistance is essential.

### 6.1. Limitations

This study confirmed that cognitive-behavioral counseling is effective in managing stress levels among administrators and general education teachers in special schools. As with all qualitative research, however, the study has limitations. The specific characteristics of the participants restrict the generalizability of the findings, and the inclusion of only 2 special schools may not fully represent all secondary schools across southeastern Nigeria. Future studies should use larger populations and sample sizes to overcome these limitations when replicating the study.

## Acknowledgments

We sincerely thank all our participants for their unwavering commitment from the beginning of the program to the end. Thank you all.

## Author contributions

**Conceptualization:** Theresa Chinma Agodo, Ngozi Sandra Ikenyesi, Vera Nneka Ogakwu, Mercy Benedette Ifeoma Uba, Ogochukwu Vivian Nwabuani, Moses Onyemaechi Ede, Beth Nnenne Oluka.

**Data curation:** Theresa Chinma Agodo, Ngozi Sandra Ikenyesi, Vera Nneka Ogakwu, Mercy Benedette Ifeoma Uba, Ogochukwu Vivian Nwabuani, Moses Onyemaechi Ede, Andrew Nnabuchi Ogbochie.

**Formal analysis:** Theresa Chinma Agodo, Ngozi Sandra Ikenyesi, Vera Nneka Ogakwu, Mercy Benedette Ifeoma Uba, Moses Onyemaechi Ede

**Funding acquisition:** Theresa Chinma Agodo, Ngozi Sandra Ikenyesi, Vera Nneka Ogakwu, Mercy Benedette Ifeoma Uba, Ogochukwu Vivian Nwabuani, Florence N. Ogbonna, Patrick Chinenye Okafor, Moses Onyemaechi Ede, Beth Nnenne Oluka, Nneka Nwosu, Andrew Nnabuchi Ogbochie, Ndubuisi Collins Enyi.

**Investigation:** Theresa Chinma Agodo, Ngozi Sandra Ikenyesi, Vera Nneka Ogakwu, Mercy Benedette Ifeoma Uba, Ogochukwu Vivian Nwabuani, Florence N. Ogbonna, Patrick Chinenye Okafor, Moses Onyemaechi Ede, Beth Nnenne Oluka, Nneka Nwosu, Andrew Nnabuchi Ogbochie.

**Methodology:** Theresa Chinma Agodo, Ngozi Sandra Ikenyesi, Vera Nneka Ogakwu, Mercy Benedette Ifeoma Uba, Ogochukwu Vivian Nwabuani, Florence N. Ogbonna, Patrick Chinenye Okafor, Moses Onyemaechi Ede, Beth Nnenne Oluka, Nneka Nwosu.

**Project administration:** Theresa Chinma Agodo, Ngozi Sandra Ikenyesi, Vera Nneka Ogakwu, Mercy Benedette Ifeoma Uba, Ogochukwu Vivian Nwabuani, Florence N. Ogbonna, Patrick Chinenye Okafor, Moses Onyemaechi Ede, Beth Nnenne Oluka, Nneka Nwosu, Andrew Nnabuchi Ogbochie.

**Resources:** Theresa Chinma Agodo, Ngozi Sandra Ikenyesi, Vera Nneka Ogakwu, Mercy Benedette Ifeoma Uba, Ogochukwu Vivian Nwabuani, Patrick Chinenye Okafor, Moses Onyemaechi Ede, Andrew Nnabuchi Ogbochie, Ndubuisi Collins Enyi.

**Software:** Theresa Chinma Agodo, Ngozi Sandra Ikenyesi, Vera Nneka Ogakwu, Mercy Benedette Ifeoma Uba, Moses Onyemaechi Ede, Andrew Nnabuchi Ogbochie.

**Supervision:** Theresa Chinma Agodo, Ngozi Sandra Ikenyesi, Mercy Benedette Ifeoma Uba, Ogochukwu Vivian Nwabuani, Florence N. Ogbonna, Patrick Chinenye Okafor, Moses Onyemaechi Ede, Nneka Nwosu, Andrew Nnabuchi Ogbochie, Ndubuisi Collins Enyi.

**Validation:** Theresa Chinma Agodo, Ngozi Sandra Ikenyesi, Mercy Benedette Ifeoma Uba, Ogochukwu Vivian Nwabuani, Florence N. Ogbonna, Patrick Chinenye Okafor, Moses Onyemaechi Ede, Beth Nnenne Oluka, Nneka Nwosu, Andrew Nnabuchi Ogbochie, Ndubuisi Collins Enyi.

**Visualization:** Theresa Chinma Agodo, Ngozi Sandra Ikenyesi, Mercy Benedette Ifeoma Uba, Ogochukwu Vivian Nwabuani, Florence N. Ogbonna, Patrick Chinenye Okafor, Moses Onyemaechi Ede, Beth Nnenne Oluka, Nneka Nwosu, Andrew Nnabuchi Ogbochie, Ndubuisi Collins Enyi.

**Writing – original draft:** Theresa Chinma Agodo, Ngozi Sandra Ikenyesi, Vera Nneka Ogakwu, Mercy Benedette Ifeoma Uba, Florence N. Ogbonna, Patrick Chinenye Okafor, Moses Onyemaechi Ede, Beth Nnenne Oluka, Nneka Nwosu.

**Writing – review & editing:** Theresa Chinma Agodo, Ngozi Sandra Ikenyesi, Vera Nneka Ogakwu, Ogochukwu Vivian Nwabuani, Florence N. Ogbonna, Patrick Chinenye Okafor, Moses Onyemaechi Ede, Nneka Nwosu, Andrew Nnabuchi Ogbochie, Ndubuisi Collins Enyi.
